# A Comparative Analysis of COVID-19 IgG Antibody Level and Socio-Demographic Status in Symptomatic and Asymptomatic Population of South Andaman, India

**DOI:** 10.7759/cureus.22398

**Published:** 2022-02-20

**Authors:** Deepak Kumar, Amrita Burma, Ashish Kumar Mandal, Vidhu Joshy

**Affiliations:** 1 Department of Community Medicine, Andaman and Nicobar Islands Institute of Medical Sciences, Port Blair, IND; 2 Department of Pathology, Andaman and Nicobar Islands Institute of Medical Sciences, Port Blair, IND; 3 Department of Biostatistics, Andaman and Nicobar Islands Institute of Medical Sciences, Port Blair, IND

**Keywords:** sero-survey, symptomatic infection, covid 19, immunity, asymptomatic infection, andaman

## Abstract

Introduction: The serosurveillance of COVID-19 antibody levels and their difference between symptomatic and asymptomatic groups can help in understanding the immune status of the community and the factors affecting it. Hence, the study was undertaken to find the differences between these two groups with respect to antibodies level and other socio-demographic variables in the South Andaman district.

Methods: A population-based serosurveillance study covering more than 4,000 samples was carried out in the South Andaman district. The participants were selected by multistage cluster sampling. The venous blood samples were tested for IgG COVID-19 antibodies by Erba Lisa Elisa kit.

Results: 5.3% of total individuals (217) were symptomatic whereas 94.7% (3,872) were asymptomatic. The symptomatic individuals had lower antibodies (33.6%) as compared to asymptomatic individuals (40.1%) (p-value=0.059). In the age group of 31-45 years, antibody positivity in the asymptomatic group was significantly higher than in the symptomatic group (p-value 0.031). The antibody positivity was higher in moderate to severe cases who needed hospital admission. The antibody positivity was found similar in both the groups in front-line workers as well as in non-front-line workers (p-value=0.104, 0.274, respectively).

Conclusion: The antibody positivity was higher in asymptomatic individuals as compared to symptomatic individuals, particularly in the age group of 31-45 years. The higher level of antibody positivity in asymptomatic individuals reflected a stronger immune response which led to no clinical manifestations. The antibody positivity was also found higher in moderate to severe cases undergoing hospital admission whereas antibodies positivity was found similar in front-line and non-front-line workers.

## Introduction

Coronavirus disease (COVID-19) emerged in De­cember 2019 and spread across the world since then [1,2]. Till February 14, 2021 over 108.2 million cases and 2.38 million deaths have been reported globally [3]. Hence, a need was felt to conduct a serosurveillance of the COVID-19 antibody among the population for understanding the immune status and the factors that affect it. This may also help policy-makers in formulating better preventive strategies for the prevention of COVID-19 in communities.

International studies from Sweden and UAE have observed the association of seroprevalence of COVID-19 antibodies with self-reports symptoms [4,5]. But, the various other seroprevalence studies conducted during the pandemic have not analyzed the differences in antibodies level in symptomatic and asymptomatic individuals [6-8]. Hence, little is known about antibodies difference between these two groups in the world and particularly in an isolated and remote island such as Andaman and Nicobar.

Andaman and Nicobar Islands are remotely located in the Bay of Bengal region and have a segregated and isolated population with its own unique demography. South Andaman district is the most developed district of the whole archipelago and has approximately 60% of the population of the whole Andaman and Nicobar islands. The whole archipelago is served by only one tertiary care hospital which is located in the South Andaman district resulting in the referral of all the cases of the island to this hospital. Hence, this study was undertaken to find the differences between symptomatic and asymptomatic cases of COVID-19 with respect to antibodies level and other socio-demographic variables in the South Andaman district.

## Materials and methods

The present study is a cross-sectional study conducted among the population of the South Andaman district. The consenting adults of 18 years and above were included in the study. The individuals suffering from any immune-deficient condition like HIV/or under chemotherapy were excluded from the study. The available literature did not provide any reliable estimates of the prevalence of COVID-19 antibodies in the Andaman and Nicobar Islands. Therefore, the sample size was calculated by considering 50% prevalence [9,10].

Further, 2.5 design effect, 2.5% absolute precision were used for sample size calculation. The sample size was calculated by the following formula:

n= [z^2^pq/d^2^] DEFF,

where n = sample size, z = linked to 95% confidence interval for cluster sampling = 2.0, p = expected prevalence (fraction of 1) = 0.5, q = 1 - p (expected non-prevalence) = 0.5, d = absolute precision = 0.025, DEFF = Design Effect = 2.5. The minimum sample size came out to be 4,000. Hence, a total of 4,089 individuals were included in the study.

The multistage cluster sampling was used for the selection of participants in the study. A village in the rural area and a municipal ward in urban areas were taken as a cluster for sampling. Based on the unique geography and demography of the island, 45 clusters (27 clusters from urban areas and 18 clusters from rural areas) were selected randomly from the approximate 125 clusters identified in the South Andaman district. In each cluster, 100 participants were selected randomly from the list of eligible residents of the cluster. There was a non-response rate of 9.2% among the total study population. The list of clusters and permission to conduct the survey was obtained from Andaman and Nicobar administration.

The first wave of COVID-19 reached its peak in August 2020 in Andaman and Nicobar Islands and it started to decline after one month [11]. Hence, the sample collection was done from December 15, 2020 to February 14, 2021 to find the status of immunity in symptomatic and asymptomatic individuals after the first wave of COVID-19 pandemic exposure on the island. The survey team collected information about the socio-demographic profiles (age, sex, geography, occupation) and medical/clinical profiles of the participants by a predesigned and pre-validated questionnaire. The survey team collected 3-5 mL venous blood samples for detecting COVID-19 antibodies. The procedure of data and blood collection was done only after the informed consent of the participants.

The health care workers were included in front-line workers and the rest of the participants were included in non-front line workers. The symptoms of participants that occurred in the past one month of survey were categorized according to guidelines issued by the All India Institute of Medical Science (AIIMS)/Indian Council of Medical Research (ICMR) COVID-19 national task force [12].

COVID-19 IgG antibodies were detected by using Erba Lisa ELISA-based test. This kit was based on the principle of indirect ELISA using recombinant Spike subunit antigen. The antibody positivity was found by calculating the antibody index. The antibody index was calculated by dividing each sample's optical density (OD) by cut-off values. The antibody index with less than 0.9 was reported as non-detectable IgG antibody for COVID-19 whereas an antibody index greater than 0.9 was reported as a detectable IgG antibody for COVID-19.

SPSS version 20.0 (IBM, Armonk, NY, USA) was used for data analysis. Descriptive analysis was done for socio-demographic variables like age, sex, residence, and occupation. The association of various categorical variables (like age, sex, residence, occupation, COVID-19 contact, hospital admission, medical consultation, previous test for COVID-19) with antibody positivity in symptomatic and asymptomatic individuals were compared with Chi-square/Fisher Exact test. The OD values of symptoms related to COVID-19 infection were analyzed with the Mann-Whitney U test. A p-value of <0.05 was considered statistically significant. The study was approved by the Institutional ethical committee of ANIIMS, Port Blair.

## Results

Among the population residing in South Andaman Islands, 4,089 persons were examined for COVID-19 antibodies. Out of this, 19.3% belonged to 18-30 years, 43.2% belonged to 31-45 years and 37.5% were above 46 years. According to gender-wise distribution, 57.0% of the population were females whereas 43.0% were males. The proportion of participants residing in urban areas was 62.9% whereas the proportion of participants residing in rural areas was 37.1%.

The symptoms of COVID-19 infections post the first wave of the COVID-19 pandemic in Andaman were present in 217 (5.3% of total) individuals whereas 3872 (94.7% of total) individuals were asymptomatic. The antibody positivity of the study individuals was reflected by the antibody index. The percentage of symptomatic people (33.6%) who were positive for antibodies was lower than asymptomatic individuals (40.1%) (Table 1).

**Table 1:** The comparison of COVID-19 antibody positivity in symptomatic and asymptomatic individuals in the South Andaman Islands.

	COVID-19 Antibody (Ab index)					Total	P-value (chi-square test)
	Positive		Negative				
	N	%	n	%			
Symptomatic	73	33.6%	144	66.4%	217		0.059
Asymptomatic	1,552	40.1%	2,320	59.9%	3,872		
Total	1,625	39.7%	2,464	60.3%	4,089		

The association of various socio-demographic variables like age, sex, geography, occupation with antibody positivity in symptomatic and asymptomatic individuals was analyzed as shown in Table 2. The antibody positivity in the symptomatic group was 28.0% as compared to 34.0% in the asymptomatic group in age groups of 18-30 years. Similarly in the age group of 31-45 years, asymptomatic individuals had significantly higher antibodies (40.9%) as compared to symptomatic individuals (29.9%) (p-value=0.031). The gender-wise analysis of antibody positivity in symptomatic and asymptomatic groups revealed that there was no significant difference in antibody positivity in symptomatic males (32.1%) as compared to asymptomatic males (38.1%) (p-value=0.269). Similarly, it was not statistically different (p-value=0.111) in symptomatic females (34.6%) as compared to asymptomatic females (41.6%).

The antibody positivity in asymptomatic rural individuals (41.1%) was also similar to symptomatic rural individuals (27.4%) (p-value=0.097). Similarly, the antibody positivity had no difference between symptomatic urban individuals (36.8%) as compared to asymptomatic urban individuals (41.9%) (p-value=0.225). The comparisons of antibody positivity in symptomatic individuals and asymptomatic individuals in relation to front-line workers did not show any significant difference (p-value=0.104). Similarly, the antibody positivity was also found similar (p-value=0.274) between symptomatic non-front-line workers (34.5%) and asymptomatic non-front-line workers (39.6%) (Table 2).

**Table 2:** The association of various socio-demographic variables and COVID-19 antibodies among symptomatic and asymptomatic individuals.

Socio-demographic Variable	Symptoms	COVID-19 Antibody				Total	P-value
		Positive		Negative			
		n	%	N	%		
Age (18-30years)	Symptomatic	14	28.6%	35	71.4%	49	0.435
	Asymptomatic	252	34.0%	489	66.0%	741	
Age (31-45years)	Symptomatic	29	29.9%	68	70.1%	97	0.031
	Asymptomatic	683	40.9%	987	59.1%	1,670	
Age (≥46years)	Symptomatic	30	42.3%	41	57.7%	71	0.997
	Asymptomatic	617	42.2%	844	57.8%	1,461	
Male	Symptomatic	27	32.1%	57	67.9%	84	0.269
	Asymptomatic	639	38.1%	1,037	61.9%	1,676	
Female	Symptomatic	46	34.6%	87	65.4%	133	0.111
	Asymptomatic	913	41.6%	1,283	58.4%	2,196	
Rural	Symptomatic	20	27.4%	53	72.6%	73	0.097
	Asymptomatic	533	37.0%	909	63.0%	1,442	
Urban	Symptomatic	53	36.8%	91	63.2%	144	0.225
	Asymptomatic	1019	41.9%	1,411	58.1%	2,430	
Front-line workers	Symptomatic	33	32.7%	68	67.3%	101	0.104
	Asymptomatic	644	40.8%	933	59.2%	1,577	
Non-front line workers	Symptomatic	40	34.5%	76	65.5%	116	0.274
	Asymptomatic	908	39.6%	1,387	60.4%	2,295	

Out of the study participants, only 5.3% (n=217) individuals had COVID-19 symptoms. The fever was the most common symptom (41%) followed by cough (35.9%) and cold (26.7%). Most of the symptoms such as fever (41%), cough (35.9%), cold (26.7%), sore throat (8.7%), myalgia (5.1%), headache (4.6%), and loss of taste (0.9%) were milder. The moderate/ severe symptoms like dyspnoea/breathing difficulty were observed only in 1.8% (n=4) of total symptomatic patients (n=217). The COVID-19 antibody level (OD value) was similar irrespective of the type of symptoms (Table 3).

**Table 3:** The association of symptoms with COVID-19 antibody (OD value). OD - optical density

History of symptoms	OD value				P-value (Mann-Whitney U test)
	Mean	Std. Deviation	Minimum	Maximum	
Fever	0.229	0.308	0	1.288	0.915
No fever	0.253	0.405	0	2.262	
Cough	0.271	0.437	0	2.262	0.211
No cough	0.227	0.323	0	1.872	
Sore throat	0.179	0.234	0	0.852	0.374
No sore throat	0.249	0.378	0	2.262	
Cold	0.257	0.456	0	2,262	0.669
No cold	0.237	0.332	0	1.872	
Headache	0.331	0.567	0	1.872	0.796
No headache	0.238	0.357	0	2.262	
Dyspnoea	0.246	0.370	0	2.262	0.632
No Dyspnoea	0.224	0.375	0	1.872	
Myalgia	0.131	0.119	0.007	0.372	0.964
No myalgia	0.248	0.376	0	2.262	
Loss of taste	0.094	0.096	0.026	0.163	0.838
No loss of taste	0.244	0.369	0.007	0.372	

The percentage positivity of antibodies in symptomatic and asymptomatic individuals with a history of contact was compared and it was found to be similar (Table 4). The seropositivity in symptomatic (32.8%) and asymptomatic individuals (40.5%) who were never tested before for COVID-19 by RT-PCR had no statistically significant difference (p-value=0.075). Similarly, the seropositivity was also compared between symptomatic (35.0%) and asymptomatic (39.1%) individuals who were previously tested at least once by RT-PCR, and it was found to be similar (p-value = 0.470). The RT-PCR tested group was further divided into two subgroups, negative and positive for COVID-19. Reviewer beta: groups. In both these subgroups, the symptomatic and asymptomatic individuals had similar percentage positivity (Table 4).

**Table 4:** The association of clinical history with COVID-19 antibodies in symptomatic and asymptomatic individuals.

Medical History	Symptoms	COVID-19 Antibody				Total	P-value
		Positive		Negative			
		n	%	N	%		
No history of COVID-19 contacts	Symptomatic	59	33.3%	118	66.7%	177	0.084
	Asymptomatic	1,455	39.8%	2,198	60.2%	3,653	
History of COVID-19 contacts	Symptomatic	14	35.0%	26	65.0%	40	0.274
	Asymptomatic	97	44.3%	122	55.7%	219	
Previously never tested for COVID-19 by RT-PCR	Symptomatic	45	32.8%	92	67.2%	137	0.075
	Asymptomatic	1,123	40.5%	1,651	59.5%	2,774	
Previously at least once tested for COVID-19 by RT-PCR	Symptomatic	28	35.0%	52	65.0%	80	0.470
	Asymptomatic	429	39.1%	669	60.9%	1,098	
Previously tested negative COVID-19 by RT-PCR	Symptomatic	19	28.8%	47	71.2%	66	0.430
	Asymptomatic	315	33.5%	625	66.5%	940	
Previously tested positive COVID-19 by RT-PCR	Symptomatic	9	64.3%	5	35.7%	14	0.531
	Asymptomatic	114	72.2%	44	27.8%	158	

To correlate the antibody level with the severity of the symptoms, antibody levels were correlated with hospital admission since only moderate to severely symptomatic cases were admitted to the hospital. The symptomatic individuals who were admitted to the hospital had higher COVID-19 antibodies (61.9%) as compared to symptomatic individuals who were not admitted to the hospital (30.6%), (p-value 0.003). However, when the treatment and medical consultation were compared between symptomatic and asymptomatic individuals, the antibody levels were found to be similar (Table 5).

**Table 5:** The association of treatment history with COVID-19 antibodies in symptomatic individuals.

	COVID-19 Antibody				Total	P-value (chi-square test)
	Positive		Negative			
	N	%	N	%		
No medical consultation	46	37.4%	77	62.6%	123	0.180
Medical consultation done	27	28.7%	67	71.3%	94	
No Hospital admission	60	30.6%	136	69.4%	196	0.003
Admitted in Hospital	13	61.9%	8	38.1	21	

## Discussion

The present study was conducted among the population of remote South Andaman Island to find the immune status of the community and the factors affecting it, whether a difference existed between the symptomatic and asymptomatic manifestation of the infection. The serosurveillance revealed that 94.7% of the individuals were asymptomatic. Similar incidences ranging from 90% to 98% were reported in the survey conducted in Delhi, Orissa, and the second round of the National Survey of ICMR [13-15]. These studies reported a higher incidence of asymptomatic individuals in respective study areas which happened due to widespread infection and asymptomatic seroconversion in the respective communities. On the contrary, the asymptomatic infections in many Asians and European countries ranged from 18% to 81%, which might have happened due to the variable density of population in these countries and variation of containment measures [16].

 When the antibody level was compared between symptomatic and asymptomatic groups then it revealed that antibody positivity was higher in asymptomatic individuals (40.1%) as compared to symptomatic individuals. The higher level of antibody positivity in asymptomatic individuals reflected a stronger immune response which subdued the infection to such a level that the case did not manifest at all [17,18]. It was further observed that in the age group of 31 to 45 years significantly higher percentage of asymptomatic individuals were antibody positive compared to the symptomatic group, probably being the working-age group they had a higher incidence of exposure. Their positivity was also higher than older individuals. This may be because at a younger age they are likely to have a more effective immune response compared to older people [19]. Further older people with comorbidities would always have decreased immunity compared to younger people [20].

In the cases that were previously RT-PCR positive compared to those who became positive later, the people who later became positive, antibody positivity was higher as compared to people who were infected previously as the time gap between these two groups was approximately 2-3 months. Hence, it may be assumed that by the time antibody was tested in the previously positive group, the antibody level had started coming down. Thus, it was lower than a recently infected group. Many studies also revealed slowly fading antibodies over a period of 14 days to 4 months in asymptomatic and mild manifestations whereas antibodies persisted comparatively for a longer duration in severe cases as an immune response was strong in them [20-24]. Further, there was no significant antibody difference between the groups which were never tested or tested at least once because majority cases suffered from asymptomatic infection and the positivity rate of RT-PCR is around 80% [14,15].

Antibody positivity was also found similar in symptomatic and asymptomatic individuals who had contact with COVID-19 positive cases as compared to symptomatic and asymptomatic individuals who had no contact with COVID-19 positive cases. The present study was done after the first pandemic but before starting of the second COVID-19 pandemic. Hence, the COVID-19 exposure and subsequent antibody development occurred due to exposure during the first wave. During this wave and a subsequent one to two months, the lockdown was very severe and strictly implemented. Public transport was completely shut down. Quarantine was strictly followed. Infected people and their contacts were kept in hotels completely isolated and away from others. Even area wise segregation and isolation were enforced. The areas were divided into three zones. The highest infectivity zone had the severest lockdown followed by the moderate infectivity zone and the rest of the areas were COVID-19 free zone. People were confined to their homes in a severe and moderate infective zone with restrictions of movement. The facemask and sanitization were strictly followed a violation was penalized. In spite of this people became seropositive across all the zones and developed a similar level of the antibody as is seen in this study. Hence the virus spread defying all the barriers. So, other than spreading through touch or close contacts there appeared to be an alternate mechanism of transmission and spread. It is known that aerosol spread can effectively bypass these mechanical barriers as they remain suspended in air over long distances and time. WHO has also suggested that aerosol is another mode of spread of this virus [25,26]. This probably was the reason that the demographic variables like gender, urban and rural divide and professional profile had no impact on infectivity and hence the antibodies level or clinical manifestations.

Similarly, the frontline workers, as well as non-front-line workers, had similar antibody levels between the symptomatic and asymptomatic group, which was in spite of the fact that their work profiles varied a lot and many did not even come in contact with any patient and they maintained all precautionary measure such as facemask, washing of hands and social distancing. This could only happen if the incidence of infection, irrespective of different factors and environmental conditions, remained similar. 

The study also revealed that no single sign and symptom of COVID-19 had any relation with levels of antibodies. In the serosurvey of Orissa, symptoms like breathing difficulty, myalgia, loss of taste/anosmia also had no association with seropositivity [14]. Though there was an association of fever, cough, and diarrhoea with the seropositivity of the participants [14]. This difference might have arisen due to the reason that the symptoms like the fever of longer duration, persistent cough, and diarrhoea are clinical manifestations of severe cases. Our study also found that moderate to severe cases which needed hospital admission had higher antibodies as compared to individuals who did not require hospital admission. Hence, the IgG type of antibody (as detected by our kit) correlated well with the severity of the manifestation. Similarly, another study that severely ill patients that were intubated or passed away due to COVID-19 had the highest levels of IgG and IgA antibodies targeting RBD and spike, but no significant differences were seen for IgM. These individuals also had the highest neutralization titers. In contrast, individuals that were not hospitalized had the lowest IgG and IgA levels and neutralization titers [27]. Few other studies also reported that neutralising antibodies titers correlated strongly with disease severity and with anti-spike IgG levels [28]. The patients from intensive care units exhibited high neutralising antibodies titers; conversely, patients with milder disease symptoms had heterogeneous neutralising antibodies titers, and asymptomatic or exclusive outpatient-care patients had no or low neutralising antibodies [8,20,21].

## Conclusions

The current study signifies that viral exposure has led to predominantly asymptomatic infection. The antibody positivity was higher in asymptomatic individuals as compared to symptomatic individuals. The higher level of antibody positivity in asymptomatic individuals reflected a stronger immune response, which led to no clinical manifestations. This was also seen in other age groups, particularly in the working-age group of 31-45 years. The antibody was seen higher in moderate to severe cases. The antibody positivity was found similar in symptomatic and asymptomatic individuals irrespective of other socio-demographic variables like gender, geography, and occupation. The antibody positivity was also found similar in individuals who had contact with COVID-19 positive individuals as compared to individuals who had no contact with COVID-19 positive individuals.
